# Petroleum in Skin Diseases

**Published:** 1876-04

**Authors:** 


Petroleum in the Treatment of Skin Diseases.—Dr. Maccormac recommends petroleum for porrigo and favus in preference to other remedies. The plan of treatment, so far as the scalp is concerned, is to clip or shave the hair closely. One part of petroleum is to be added to two of lard. This ointment should be applied gently but thoroughly once or twice a day. In cases that have been neglected, poultices of bread or linseed meal should be used before applying the ointment. After the application, a piece of dry, soft linen rag may be laid on, and over all a clean linen cap. Before the next application of the ointment, the head must be cleansed with black or fish soap, and warm water.—*The Practitioner*, October, 1875.

